# Microbial dormancy as an ecological and biogeochemical regulator on Earth

**DOI:** 10.1038/s41467-025-59167-6

**Published:** 2025-04-25

**Authors:** James A. Bradley

**Affiliations:** 1 Aix Marseille Université, Université de Toulon, CNRS, IRD, MIO, Marseille, France; 2 School of Biological and Behavioural Sciences, Queen Mary University of London, London, UK

**Keywords:** Biogeochemistry, Microbial ecology, Environmental sciences, Environmental microbiology

## Abstract

Virtually all of Earth’s ecosystems and biogeochemical cycles are underpinned – and often driven – by the activity (or inactivity) of microorganisms. Dormancy, a reversible state of reduced metabolic activity, is ubiquitous among microbial communities in environments ranging from moderate to extreme. Dormancy enables microorganisms to withstand severe and widespread environmental changes. Here I argue that dormancy exerts a powerful influence on Earth’s ecological and biogeochemical architecture through space and time, and over vast scales. Dormancy manifests differently across taxonomically and functionally distinct microbial groups, and operates over timescales ranging from hours to millennia – enabling microorganisms to interact with the geosphere over geologically relevant timescales. As such, dormancy may play a crucial role in shaping ecosystems and biogeochemical cycles throughout the Earth system. Interdisciplinary, integrative geosphere-biosphere approaches will be essential for advancing our understanding of how microbial dormancy underpins the co-evolution of Earth, its biosphere, and their interactions.

## Abstract (web summary)

Dormancy enables microbes to withstand periods of environmental harshness. It occurs throughout diverse ecosystems and spans vast timescales, and could play an important role in shaping the co-evolution of Earth’s biosphere and geosphere.

## Introduction

Microorganisms are significant drivers and regulators of virtually all major elemental cycles on Earth, impacting the surface redox state of the planet and global climate^1^. The Earth has changed profoundly throughout geological time: orbital variabilities^2^ and tectonic processes^3,4^ have caused climate, geography, and the composition of the atmosphere and the oceans to shift—resulting in substantially different temperature, light, radiation, and redox conditions. Microorganisms have evolved to occupy virtually all available environmental niches on Earth’s surface and near-surface envelopes, acquiring adaptations to thrive within or tolerate specific ranges in environmental conditions^5^. When environmental conditions change, certain microbial groups can be subject to extinctions^6,7^. Nevertheless, despite widespread and extreme environmental changes on Earth, occurring over short (e.g., seasonal) to geological timescales (including global glaciations lasting millions of years^6,8–10^), microorganisms have continued to accrue distinct phylogenies, morphologies, physiologies, and functions^6,11^. The adaptive capacity of microorganisms enables them to thrive throughout environmental changes. This makes them unique objects of study for understanding the co-evolution of Earth and its biosphere.

**Fig. 1:**
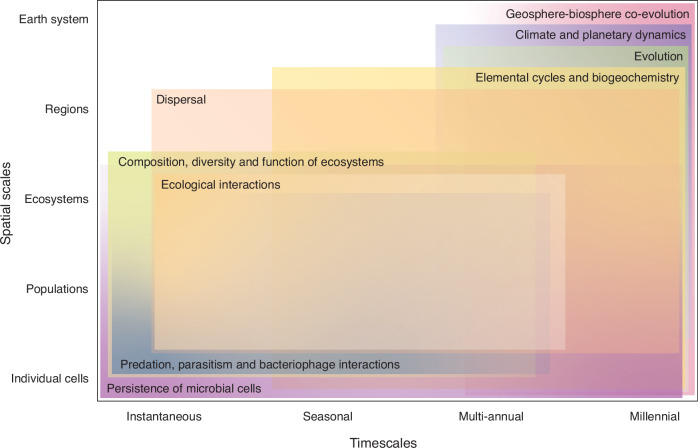
Microbial dormancy and its effects through time and space. This diagram illustrates the various processes and phenomena that are shaped and impacted by microbial dormancy, and the timescales (from immediate to millennial) and spatial scales (from individual cells to the Earth system) over which dormancy may have an influence. Different processes and phenomena are distinguished by hue, and gradients in opacity represent dormancy exerting a strong (more opaque) to weak (more transparent) influence.

**Fig. 2:**
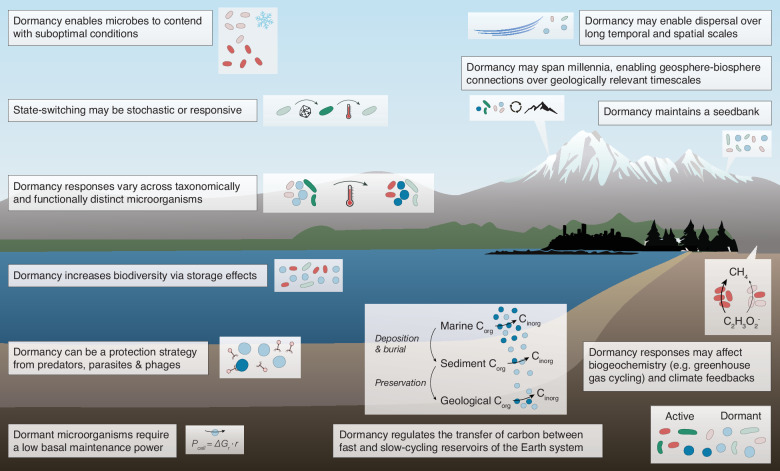
Microbial dormancy and its effects throughout the Earth system. Levels of dormancy and activity of individual microbial cells and communities of microorganisms are sensitive to ecological and biogeochemical characteristics, processes, and alterations. Likewise, microbial dormancy may be immensely influential on ecosystem dynamics, biogeochemical cycles and Earth system processes. This conceptual diagram illustrates microbial dormancy and its effects through and across the Earth system and its components. Taxonomically and functionally distinct microbial lineages are distinguished by different hues. Dormant cells are indicated by lighter tints and active cells are indicated by darker shading.

Microorganisms use dormancy as a strategy to persist in harsh conditions and throughout unfavorable changes in their environment. Dormancy is a reversible state of reduced metabolic activity, and it enables microbes to contend with environmental harshness including limited energy and resource availability, pressure, temperature, and other biotic and abiotic factors that fluctuate over space and time^12^. Dormancy is ubiquitous among microbial communities in environments that are moderate to extreme, oligotrophic to eutrophic, and low in diversity to highly biodiverse^12^. It is a strategy by which microorganisms engaging in dormancy withdraw from being active players in the present environment and become part of a seedbank—prolonging their persistence, contributing to community dynamics in future ecosystems, and maintaining biodiversity over time. In this work, I argue that microbial dormancy is of particular interest for understanding Earth’s ecological and biogeochemical architecture through space and time across vast scales. Levels of dormancy, activity and state-switching across individual cells and communities of microorganisms are highly variable, and are sensitive to ecological and biogeochemical characteristics, processes, and alterations. Microbial dormancy and its effects transcend vast spatial scales from individual cells to the Earth system, and span timescales from instantaneous responses, to seasonal, millennial and even geologic timescales (Fig. 1). Dormancy is prevalent throughout taxonomically and functionally diverse microbial lineages, and across multiple interacting components of the Earth system (Fig. 2). If certain functionally or taxonomically distinct microbial groups are trigged into or out of dormancy by particular environmental cues, this may bring about changes to ecosystem functioning, biodiversity, and biogeochemical cycles. As we continue to uncover these connections, we will gain new insights into how microbial dormancy shapes and drives the co-evolution of Earth’s geosphere and biosphere.

## Dormancy in the context of geobiology and Earth system science

Dormancy is widespread often characterises the majority of microbial cells in natural settings^13^. Some microorganisms produce visibly distinct resting structures such as spores or cysts when engaging in dormancy, whereas other dormant microorganisms are morphologically indistinguishable from active cells. Dormancy may arise due to environmental cues such as light, temperature, and resource availability that are suboptimal for growth and reproduction. Dormancy can also arise from endogenous factors including species interactions, suggesting that dormancy can serve as protection from predators and parasites^12,13^. While in a dormant state, some microorganisms maintain functioning of the sensory systems necessary to detect and respond to favorable changes in their environment^14,15^. Sensing of the extracellular environment enables ‘responsive switching’—a behavior that permits microorganisms to quickly take advantage of the onset of favorable conditions by resuming a high activity state or growth^16^, and this responsive switching is thought to be a favored trait in environments experiencing fluctuating conditions. Alternatively, ‘spontaneous switching’ is a phenomenon whereby microorganisms stochastically reduce or initiate metabolic activity to enter or exit dormancy randomly. By spontaneously becoming dormant, some portion of the population forgo the opportunity to reproduce in the present setting, but are subject to reduced mortality—which would be advantageous if the environment is harsh or unfavorable for growth^15^. Spontaneous switching is thought to be favored in stable or unpredictable environments^17^.

Dormancy enables a population to maintain a reservoir of microbial diversity (i.e., a seedbank) which might otherwise be lost due to local extinctions^13^ and competition^18^. Dormancy therefore plays an important role in the resilience of microbial biodiversity and functions in the face of local to global-scale environmental change^19^. The effective ‘storage’ of dormant individuals within a seedbank also increases the biodiversity that can be harbored within a given environment by temporally partitioning ecological niches and preventing the competitive exclusion of species^20–22^. Dormancy, in some cases, also weakens antagonistic interactions^23^ and stabilizes mutualistic interactions^24^. Laboratory studies and modeling simulations have demonstrated that repeated transitions into and out of dormancy enable high levels of phenotypic and genetic diversity to be maintained over time^25–27^. Dormancy may confer other ecological advantages including enabling long-distance dispersal^28^, predator avoidance^29,30^, reducing rates of parasitic infection^31^, and safeguarding of microbial population sizes and their phenotypic and genetic diversity from bacteriophages^27,31–33^. Dormancy has also been implicated in evolution, where populations with a high fraction of dormant individuals are subject to weaker selection pressures, lower frequencies of mutations, and reduced effects of genetic drift^26^.

The enormous diversity of microorganisms, coupled with their occupation of a wide array of environmental niches, implies that dormancy strategies and responses are heterogeneous across functionally and taxonomically distinct microbial groups. Microbial dormancy may therefore play key roles in regulating elemental cycles and the biogeochemical architecture of ecosystems and the various components of the Earth system. Early studies reasoned that dormant microorganisms are passive, non-contributory players in soil biogeochemical cycling, with processes like soil organic carbon remineralization and nutrient cycling steered largely by the active fraction of microbial communities^34,35^. Consequently, Earth system models that represent microbial communities as a whole (i.e., without distinguishing between active and dormant groups) may not be able to adequately capture and quantify microbially driven carbon transformations and other element cycles^36,37^. This limitation is particularly salient in highly dynamic environments, such as in high latitude settings that experience pronounced seasonal changes—where dormancy transitions triggered by environmental changes may substantially alter the intensity of biogeochemical cycles over time^38–40^. Functionally diverse microorganisms may be triggered into activity or dormancy by a multitude of varying environmental cues occurring over a wide range of timescales. Considering this, I hypothesize that dormancy responses dampen, intensify, and alter dominant biogeochemical transformations occurring within and across environments, and change oxidation/reduction (redox) states and supplies of nutrients. However, studies directly linking microbial state-switching to shifts in biogeochemistry and elemental cycles across large temporal and spatial-scales (i.e., geologic time and global scales) are lacking. Nevertheless, it is known that dormant microorganisms play important roles in regulating critical global-scale biogeochemical cycles and ecosystem services, such as atmospheric gas concentrations^41,42^. Moreover, even microorganisms subsisting under extreme energy limitation are vastly influential on global biogeochemical cycles when framed over long timescales. For instance, microbes in marine sediments subsist at the lowest power utilization (i.e., rate of energy use) known to all life^43^, and thus are likely to be mostly dormant rather than growing—yet they degrade enormous quantities of organic carbon^44,45^ and thereby regulate the transfer of carbon between the fast-cycling and slow-cycling portions of the global carbon cycle^46^, affecting Earth’s climate and oxygenation^47,48^.

Despite the suspected pervasiveness of dormancy among microbial systems, there are major gaps in dormancy theory, and we lack a precise understanding of the wider role that dormancy might play in regulating Earth system processes, including the development of Earth’s biosphere and biogeochemical cycles. Despite recent advances in measuring how dormancy manifests among microbial communities in the laboratory and in nature, there is relatively little known in the broader context of how dormancy might enable the persistence of life and maintenance of biodiversity throughout environmental changes, especially over longer timescales such as through major climatic transitions on Earth. Little is also known about whether the activities of certain biogeochemically-relevant metabolic functions are disproportionately affected by dormancy responses, or the sensitivity of state-switching behaviors to environmental triggers across metabolically diverse microbial lineages. We therefore lack an appreciation of the sensitivity of local and global elemental cycles to microbial dormancy responses. Moreover, the precise temporal and energetic limits to dormancy, although vast, are not known. Dormant and non-growing microorganisms must uphold a minimum power requirement that is necessary for carrying out essential physiological functions over time, such as maintaining membrane integrity and regenerating enzymes^49–51^. These ‘maintenance’ activities constitute the sum of activities that do not produce growth, and are lower for microorganisms undergoing dormancy than for active or growing microorganisms. Early attempts to quantify the minimum cell-specific power required to sustain microbial cells relied mainly on studying cell cultures grown in chemostatic chambers, and produced estimates of 10^−15^ to 10^−14 ^W per cell^49,51^. However, measurements of maintenance energy demand that are derived from pure or mixed cultures of microorganisms undergoing laboratory-induced starvation cannot be readily extrapolated to natural ecosystems, and it was later shown that microorganisms in natural environments, including the vast subseafloor biosphere, subsist at cell specific powers that are many orders of magnitude lower (10^−19^ to 10^−17 ^W per cell)^52–55^, and indeed lower than what was previously shown to be the minimum power utilization to support microbial life on Earth (<10^−20 ^W per cell)^43^. Despite enduring prolonged and extreme energy limitation, dormant microorganisms may survive over millennia^56,57^, thereby enabling them to interact with Earth’s geosphere over geologically relevant timescales^58^.

## Advances and future steps

The activity or dormancy of microorganisms can determine the role that individual cells and communities play in ecological systems and processes, as well as in elemental cycles. Developing this proposition, I reason that any trigger—natural or anthropogenic—that brings about a transition of microbes between active and dormant states, may profoundly affect ecosystem dynamics, the functioning of various components of the Earth system, and their biogeochemistry. However, there is a sparseness of knowledge on the prevalence, triggers, and timescales of dormancy, that are necessary to assess these connections. Addressing this research area will require innovative and interdisciplinary approaches combining recent advances in biological and Earth sciences and state-of-the-art tools spanning molecular biology, genomics, and numerical modeling. Methodological and technological developments in cell labeling based on activity, and the visualization and sorting of active and inactive microbial cells, coupled with meta-omics and bioinformatic advances, now enable the activity and dormancy of microorganisms to be determined at the scale of individual cells within complex communities and natural environments^41,59–62^. Knowledge of the physiological state and sensitivities of individual cells in their native environment is crucial to determine the roles that the activity or inactivity of these cells play in complex ecosystems and in specific elemental transformations and biogeochemical cycles. This knowledge is opportune for integration into the next generation of numerical models, which specifically resolve microbial traits, activity and dormancy states, and functions, across scales spanning individual cells to ecosystems to Earth systems^37,63^. Embedding dormancy theory into Earth system science will provide a new lens through which to evaluate how microbial communities and their environments co-evolve, whilst contributing new tools to measure, understand, and manipulate microbial dormancy in the lab and across diverse ecosystems. The knowledge of how dormancy transforms and shapes ecosystems, biodiversity, redox states, and biogeochemistry of various environments can be applied forwards in time, for example to forecast and manage the impacts of climate change on ecosystems and elemental cycles. Dormancy theory and knowledge can also be applied backwards in time, for instance, to assess how past prolonged and widespread changes in Earth’s ecosystems, elemental cycles, and climate, have been regulated by microorganisms.

Understanding the interplay between microbial dormancy, ecological processes and biogeochemical cycles is critical to understanding various facets of the co-evolution of life and our planet, and how life persists and excels in extreme environments and throughout global environmental changes, including glacial-interglacial cycles. By elucidating the relationship between dormancy, microbial ecology, and biogeochemistry, we have the potential to change the paradigm that the fitness of organisms inhabiting hostile environments, or in fact any environment, is determined by their growth performance under favorable conditions. To the contrary, advancing theory and knowledge of microbial dormancy could demonstrate that the persistence of microbial cells through unfavorable conditions is as important as growth responses to favorable conditions. This would call for an overhaul in the way microbial ecosystems are studied, putting a spotlight on survival and persistence during periods of environmental disturbance and harshness rather than on the characterization of growth dynamics (as is typical of many microbiological investigations). Interdisciplinary and integrated approaches spanning biology to Earth systems science will be crucial to advancing knowledge of how microbial dormancy underpins the co-evolution of Earth, its biosphere, and their interactions.
